# Severity-graded supportive care need profiles in older adults during recovery after ischemic stroke: a latent profile analysis

**DOI:** 10.3389/fpubh.2026.1940952

**Published:** 2026-09-14

**Authors:** Hao Bai, Kuanhong Shen, Yating Zhan, Li Dai, Yuanzhi Li, Weidong Gong, Zhenfa Li, Jun Yin, Feng Lu, Xiaochun Tang, Jinsheng Tan, Panfeng Li, Li Liao, Zhangyi Wang

**Affiliations:** 1 School of Nursing, University of South China, Hengyang, China; 2 Orthopedic Center / Gastrointestinal Surgery / National Key Clinical Construction Specialist General Surgery, Affiliated Hengyang Hospital of Hunan Normal University & Hengyang Central Hospital, Hengyang, Hengyang, Hunan Province, China

**Keywords:** ischemic stroke, latent profile analysis, older adults, post-stroke care, stroke recovery, supportive care needs

## Abstract

**Background:**

Supportive care needs may vary substantially among older adults during recovery after ischemic stroke and are relevant to the continuity of post-stroke rehabilitation and follow-up care. A person-centered approach may help characterize heterogeneity in need intensity and inform needs-based supportive care planning. However, evidence on supportive care need profiles and their associated factors in this population remains limited.

**Methods:**

A cross-sectional study was conducted among 388 older adults with ischemic stroke recruited from three tertiary hospitals in China. Supportive care needs were assessed using the Stroke Supportive Care Needs Scale. Latent profile analysis was performed using the mean scores of six supportive care need domains. Multinomial logistic regression was used to examine factors cross-sectionally associated with latent profile membership.

**Results:**

Three severity-graded profiles were identified: the low supportive care needs group (Class 1, *n* = 120, 30.9%), moderate supportive care needs group (Class 2, *n* = 142, 36.6%), and high supportive care needs group (Class 3, *n* = 126, 32.5%). Disease-related information needs were prominent across all profiles, whereas life care, symptom management, and rehabilitation care needs were particularly elevated in the high-need profile. Compared with Class 1, older age, anxiety symptoms, and depressive symptoms were associated with higher odds of membership in both Class 2 and Class 3, whereas better self-care ability was associated with lower odds of membership in these higher-need profiles. Higher educational attainment was generally associated with lower odds of higher-need profile membership, while higher income was associated with lower odds of Class 3 membership.

**Conclusion:**

Older ischemic stroke survivors during recovery exhibited severity-graded heterogeneity in supportive care needs. Membership in the higher-need profiles was cross-sectionally associated with older age, poorer self-care ability, anxiety symptoms, depressive symptoms, and lower educational attainment, while higher income was associated with lower odds of membership in the high-need profile. These findings provide descriptive evidence for needs-based assessment and the future development of tiered rehabilitation and follow-up support during stroke recovery. Further longitudinal and external validation is needed to examine the stability and potential application of these profiles in post-stroke care.

## 1 Introduction

Stroke remains a major public health challenge and one of the leading causes of disability and dependency worldwide (1). Ischemic stroke accounts for the majority of stroke cases, with older adults disproportionately affected (2, 3). With population aging and advances in acute stroke treatment, an increasing number of older adults survive the acute event and enter an extended period of recovery (4, 5). During this period, they may experience persistent physical impairments, difficulties in symptom management and daily functioning, emotional distress, and ongoing rehabilitation needs (6). These challenges extend beyond acute clinical treatment and require coordinated support across hospital, family, primary care, and community settings. Consequently, identifying and addressing supportive care needs is relevant not only to individual recovery but also to the continuity and organization of post-stroke care (7).

Supportive care needs refer to the multidimensional assistance required by patients in response to physical, informational, psychological, social, daily living, and rehabilitation-related challenges during recovery (8). Previous studies have shown that stroke survivors frequently report unmet needs across several of these domains (9). Such needs may be particularly relevant among older adults, who may experience greater functional limitations, care dependency, and continuing rehabilitation demands after stroke (10). Anxiety and depressive symptoms are also common following stroke and may coexist with greater needs for psychological, informational, and practical support during recovery (11, 12). Taken together, supportive care needs may reflect the combined influence of functional status, psychological symptoms, and the personal and social circumstances in which recovery occurs.

However, supportive care needs are not uniformly distributed among stroke survivors (13). Most previous studies have adopted variable-centered approaches, focusing on average need scores or associations between individual characteristics and supportive care needs (14). Although these approaches are informative, they may provide limited insight into whether multidimensional need intensity clusters differently across individuals. Latent profile analysis (LPA) is a person-centered, model-based approach that can identify subgroups of individuals with similar response patterns across multiple continuous indicators (15). Rather than replacing continuous need scores, LPA may provide a complementary description of within-population heterogeneity and offer a descriptive basis for developing tiered needs assessment and follow-up strategies.

Nevertheless, evidence remains limited regarding supportive care need profiles among older adults during early recovery after first-ever ischemic stroke. The demographic, socioeconomic, functional, and psychological characteristics associated with profile membership also remain insufficiently understood. Therefore, this study aimed to identify latent profiles of supportive care needs in this population and to examine the cross-sectional associations between participant characteristics and profile membership. The findings were intended to provide descriptive evidence that may inform the future development and evaluation of needs-based supportive care assessment and follow-up strategies.

## 2 Methods

### 2.1 Study design and participants

This cross-sectional study recruited participants using convenience sampling from the neurology departments of three tertiary Grade A hospitals affiliated with the University of South China in Hengyang, Hunan Province, China, between November 2025 and March 2026. This study was reported in accordance with the Strengthening the Reporting of Observational Studies in Epidemiology (STROBE) statement for cross-sectional studies (Supplementary Table 1).

Eligible participants met the following criteria: (1) age ≥60 years; (2) first-ever ischemic stroke confirmed by computed tomography (CT) or magnetic resonance imaging (MRI) in accordance with the Chinese Guidelines for the Diagnosis and Treatment of Acute Ischemic Stroke (16); (3) being in the recovery phase (15 days to 6 months) (17); (4) having stable vital signs; (5) able to understand and respond to the questionnaire, with assistance if needed; and (6) provision of written informed consent.

Exclusion criteria were: (1) severe hepatic or renal insufficiency, malignant tumors, or other serious comorbidities; and (2) severe cognitive impairment or a documented history of major psychiatric disorders that would prevent participants from understanding the questionnaire or providing reliable responses.

The sample size was estimated primarily according to the requirements of LPA. Previous methodological research has suggested that a sample size of at least 300 participants is generally recommended to obtain stable classification in LPA (18). Considering a potential 10–20% rate of incomplete or invalid questionnaires, at least 330–375 participants were required. A total of 388 participants were ultimately included in the final analysis, which met the recommended sample size requirement for LPA.

### 2.2 Measures

#### 2.2.1 General demographic and clinical characteristics

Participants’ demographic, clinical, and health-related characteristics were collected using a self-designed information questionnaire (Supplementary Figure 1). Demographic variables included age, sex, educational level, marital status, monthly income, place of residence, and living arrangement. Clinical and health-related variables included height, weight, family history of stroke, regular physical exercise, smoking status, drinking status, number of chronic diseases, and time after stroke. Body mass index (BMI) was calculated from measured height and weight.

Age, BMI, and time after stroke were analyzed as continuous variables. Time after stroke was defined as the interval, in days, from stroke onset to questionnaire completion. Sex was categorized as male or female. Educational level was grouped into primary school and below, junior/high school, and above high school. Marital status was categorized as married or unmarried/divorced/widowed. Monthly income referred to the participant’s individual monthly income and was categorized as <4,000 CNY, 4000–5,999 CNY, and ≥6,000 CNY. Place of residence was classified as rural or urban. Living arrangement was categorized as living with family or other household members, living in a nursing home, or living alone. Family history of stroke was categorized as yes or no.

Regular physical exercise was defined as engaging in physical activity for at least 30 min at least once per week, including walking, rehabilitation exercise, or other forms of exercise. Smoking status and drinking status were categorized as current use versus no/former use. Chronic diseases included diabetes mellitus, hypertension, coronary heart disease, chronic respiratory diseases, and other physician-diagnosed chronic conditions. The number of chronic diseases was categorized as 0, 1–2, and ≥3.

#### 2.2.2 Supportive care needs scale

Supportive care needs were measured using the Stroke Supportive Care Needs Scale developed by Yu and colleagues (19). The questionnaire includes 34 items across six domains. Each item is rated on a 5-point Likert scale from 0 (“not at all”) to 4 (“extremely needy”). Domain mean scores were further calculated by dividing each domain score by the number of items in that domain, with higher scores indicating greater supportive care needs. Previous studies have reported good psychometric properties, with a total Cronbach’s alpha of 0.937; in the current sample, Cronbach’s alpha was 0.881.

#### 2.2.3 Geriatric Depression Scale-15 (GDS-15)

The GDS-15 is a simplified version of the original Geriatric Depression Scale developed by Sheikh (20) following an in-depth evaluation of the full instrument. The scale consists of 15 items with dichotomous “yes/no” responses. For most items, a response of “yes” is scored as 1 and “no” as 0; however, Items 1, 5, 7, and 11 are reverse-scored, with “no” scored as 1. The total score ranges from 0 to 15, with a score ≥8 indicating depressive symptoms. Higher scores indicate more severe depressive symptoms. Previous studies have reported acceptable reliability for the GDS-15, with Cronbach’s α of 0.82. In the current sample, Cronbach’s alpha was 0.936.

#### 2.2.4 Generalized anxiety disorder-7 (GAD-7)

The GAD-7 was developed by Spitzer et al. (21) It is used to assess the severity of anxiety symptoms. The scale consists of seven items, each rated on a 4-point Likert scale. The total score ranges from 0 to 21, with a score of ≥5 indicating the presence of anxiety symptoms; higher scores reflect greater severity of anxiety. Previous studies have reported good reliability for the GAD-7, with Cronbach’s α of 0.842. In the current sample, Cronbach’s alpha was 0.794.

#### 2.2.5 The Barthel Index (BI)

The BI (22) was used to assess participants’ ability to perform basic activities of daily living. The total score ranges from 0 to 100, with higher scores indicating better self-care ability. Referring to previous stroke supportive care needs research, BI scores were categorized into three groups: ≤40, 41–60, and 61–99 for subsequent analyses. The scale has shown good reliability in previous studies, with Cronbach’s alpha of 0.929; Cronbach’s alpha in the current sample was 0.909.

### 2.3 Pilot study and data collection

Data collection was conducted in the neurology departments of three tertiary Grade A hospitals affiliated with the University of South China in Hengyang, Hunan Province, China. Prior to the formal investigation, a pilot survey was carried out in November 2025. Twenty older adults with ischemic stroke were recruited by convenience sampling according to the same predefined eligibility criteria to evaluate whether the questionnaires were understandable and feasible for use in the target population. These participants were not included in the final analysis. Feedback obtained during the pilot survey was used to refine the wording, sequence, and layout of the questionnaires.

For the formal investigation, all exclusions shown in Supplementary Figure 2 occurred during initial eligibility screening before enrolment and data collection. During both the pilot survey and the formal data collection, trained researchers introduced the study objectives, procedures, and confidentiality arrangements to eligible participants, and written informed consent was obtained before questionnaire completion. Participants were encouraged to complete the questionnaires independently whenever possible. When participants were unable to complete the questionnaires on their own because of poor vision, limited literacy, writing difficulty, limb weakness, or fatigue, trained investigators explained the items using clear and neutral language and recorded the participants’ answers without providing prompts or leading responses. Thus, all questionnaire responses reflected the participants’ own reports. After completion, questionnaires were reviewed on site, and any missing responses were clarified with participants before final submission. The collected data were entered into a secure database and independently checked for accuracy. Following these on-site checks and clarifications, no item-level missing data remained in the final dataset. All 388 participants had complete data for the variables included in the main analyses; therefore, no imputation was conducted, and no participants were excluded from the analyses because of missing data.

## 3 Statistical analysis

Analyses were conducted using Mplus version 8.3 and R version 4.5.1. Continuous variables were described as mean ± standard deviation (SD) when normally distributed or as median and interquartile range (IQR) when non-normally distributed. Categorical variables were presented as frequencies and percentages. Because the main study variables were collected primarily through questionnaire-based assessment, common method bias was assessed using Harman’s single-factor test (23). This test examines whether a single factor accounts for a substantial proportion of the covariance among measured variables, which may indicate potential bias attributable to the measurement method rather than to the constructs of interest.

Latent profile analysis (LPA) was performed to identify supportive care need profiles among older adults with ischemic stroke. LPA is a person-centered, model-based approach used to identify unobserved subgroups of individuals with similar response patterns across continuous indicators (15). In this study, participants were classified according to the mean scores of the six domains of the Stroke Supportive Care Needs Scale. Models with one to five latent profiles were estimated. Model selection followed published recommendations for mixture modeling and considered multiple complementary criteria, including model fit, classification quality, class size, parsimony, and clinical interpretability (24, 25). Lower Akaike information criterion (AIC), Bayesian information criterion (BIC), and adjusted Bayesian information criterion (aBIC) values indicated better relative fit, whereas significant Lo–Mendell–Rubin likelihood ratio test (LMR-LRT) and bootstrap likelihood ratio test (BLRT) results indicated that a k-profile model fitted better than a k − 1 profile model. Entropy and average posterior probabilities were used to assess classification quality, with higher values indicating clearer classification. Adequate class size, parsimony, and clinical interpretability were also considered to avoid over-extracted or clinically nonmeaningful solutions. The final model was selected by balancing these statistical and substantive criteria rather than relying on any single fit index.

After the optimal latent profile model was selected, participants were assigned to the profile with the highest posterior probability. Group differences in demographic, clinical, functional, and psychological characteristics across latent profiles were examined using one-way analysis of variance or the Kruskal–Wallis test for continuous variables, and the chi-square test or Fisher’s exact test for categorical variables, as appropriate.

Multinomial logistic regression was then used to examine factors associated with latent profile membership, with the low supportive care needs profile as the reference group. Variables showing statistically significant differences across latent profiles in the univariate analyses were considered for inclusion in the multivariable model. To improve model stability and avoid overadjustment, the final covariates were selected by considering statistical significance in univariate analyses, clinical relevance, prior evidence, and avoidance of redundant or outcome-proximal variables. For the multinomial logistic regression, living arrangement was dichotomized as living with family or others versus not living with family or others because of the small numbers in the nursing home and living-alone categories. Multicollinearity among independent variables was assessed using the generalized variance inflation factor(GVIF), with adjusted GVIF values used for variables with multiple degrees of freedom. Odds ratios (ORs) and 95% confidence intervals (CIs) were reported. All tests were two-sided, and *p* < 0.05 was considered statistically significant.

## 4 Ethical considerations

This study was conducted in accordance with the Declaration of Helsinki and relevant guidelines and regulations. The study was approved by the Ethics Committee of the University of South China (approval number: 2025NHHL140). All participants were informed of the study purpose and procedures, and participated voluntarily. Written informed consent was obtained before data collection. Participants’ privacy and confidentiality were strictly maintained, and all data were anonymized and used solely for research purposes.

## 5 Results

A total of 388 older adults with ischemic stroke were included in the analysis, and the participant selection process is shown in Supplementary Figure 2. The median age was 70.00 years (IQR: 65.00–75.00), 54.4% were male, and the median time after stroke was 18.00 days (IQR: 17.00–24.00). Detailed demographic and clinical characteristics are presented in Supplementary Table 2.

### 5.1 Assessment of common method bias

Harman’s single-factor test indicated that the first principal factor explained 28.99% of the total variance, suggesting that common method bias was not substantial.

### 5.2 Latent profile analysis

Latent profile models with one to five classes were fitted to determine the optimal classification of supportive care needs (Table 1). AIC and aBIC decreased as the number of classes increased, whereas BIC decreased through the four-class model and increased in the five-class model. The LMR-LRT was not significant for the four- and five-class solutions (*p* = 0.2563 and *p* = 0.3923, respectively), indicating that the four-class model did not significantly improve fit over the three-class model and that the five-class model did not significantly improve fit over the four-class model. Entropy also decreased in the four- and five-class models (0.840 and 0.819, respectively). Although the BLRT remained significant across all models, the LMR-LRT results, classification accuracy, parsimony, and clinical interpretability supported selection of the three-class solution.

**Table 1:** Model fit results for the latent profile analysis of supportive care needs (*N* = 388).

Classes	AIC	BIC	aBIC	Entropy	LMR-LRT (p)	BLRT (p)
1-class	13287.844	13335.376	13297.301	–	–	–
2-class	12198.846	12274.105	12213.819	0.916	<0.001	<0.001
3-class	11798.977	11901.963	11819.467	0.906	<0.001	<0.001
4-class	11761.202	11891.916	11787.209	0.840	0.2563	<0.001
5-class	11747.187	11905.627	11778.710	0.819	0.3923	0.0128

The three-class model showed high entropy (0.906), significant LMR-LRT and BLRT results (both *p* < 0.001), and adequate class sizes. Although the information criteria continued to decrease in the four- and five-class solutions, their LMR-LRT results were not significant and their entropy values were lower. Considering classification quality, parsimony, and substantive interpretability, the three-class solution was selected. The average posterior probabilities for the three classes were all above 0.90 (0.980, 0.943, and 0.948 for Classes 1, 2, and 3, respectively), indicating relatively clear classification separation (Table 2).

**Table 2:** Average posterior probabilities for the three latent profiles (*N* = 388).

Class	C1	C2	C3
C1	0.980	0.020	0.000
C2	0.017	0.943	0.041
C3	0.000	0.052	0.948

Based on the three-class model, Class 1 (*n* = 120, 30.9%) had the lowest levels across all six supportive care need dimensions and was labeled the “low supportive care needs group.” Class 2 (*n* = 142, 36.6%) showed moderate levels of supportive care needs overall and was labeled the “moderate supportive care needs group.” Class 3 (*n* = 126, 32.5%) had the highest levels across all dimensions and was labeled the “high supportive care needs group.” Across the three classes, disease-related information needs were consistently among the most prominent domains, and participants in Class 3 also showed particularly high needs for life care, symptom management, and rehabilitation care. Detailed domain scores across the three classes are presented in Supplementary Table 3.

Figure 1 illustrates the mean scores for the six supportive care need domains across the three classes, showing that the profiles mainly differed in overall severity rather than in qualitatively distinct configurations.

**Figure 1:**
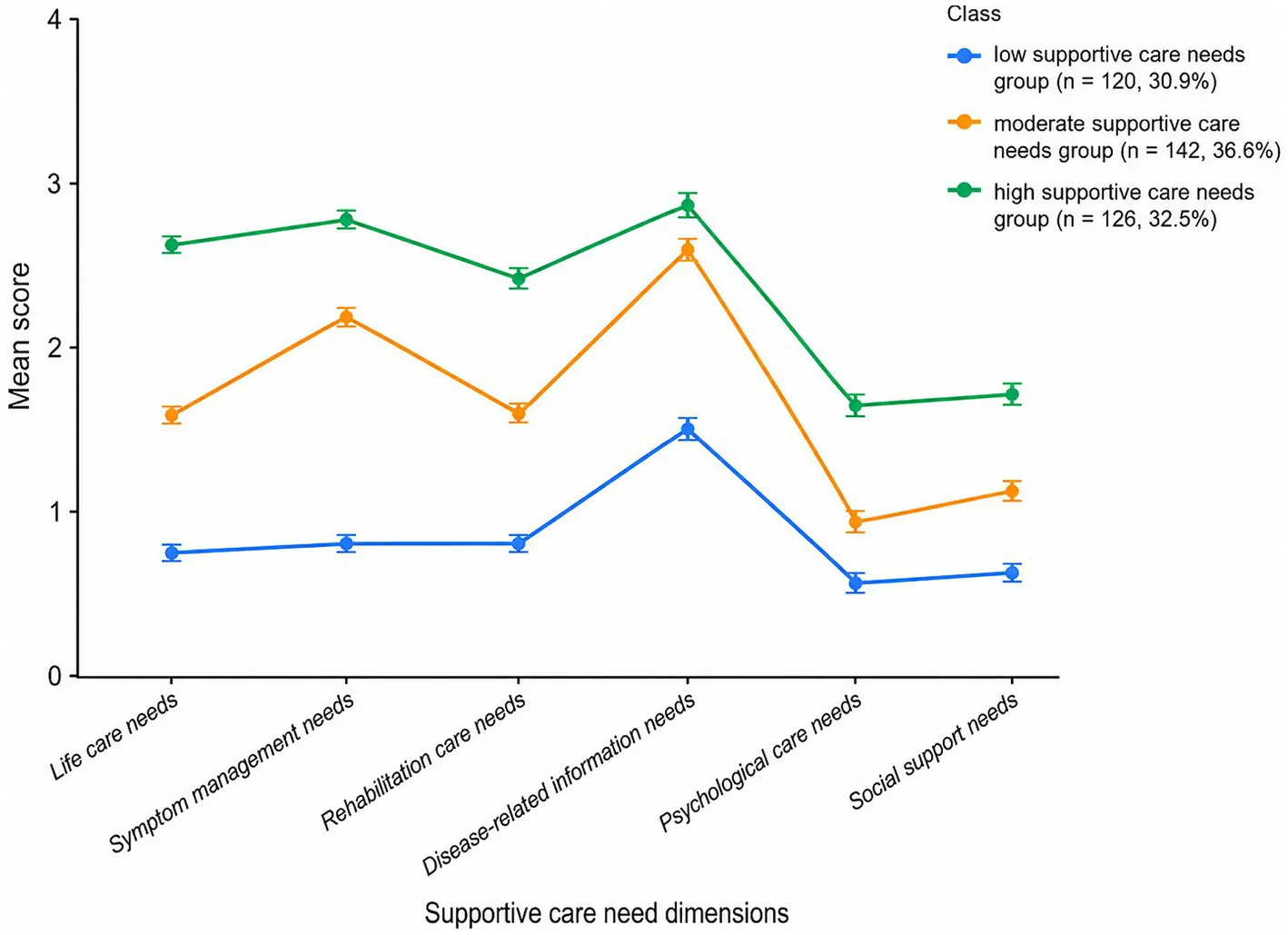
Mean scores of the six supportive care need domains across the three latent profiles.

### 5.3 Univariate analysis of factors associated with latent profile membership

Univariate analysis showed significant differences across the three latent profiles in age, BMI, sex, educational level, marital status, monthly income, living arrangement, self-care ability, number of chronic diseases, anxiety symptoms, and depressive symptoms (all *p* < 0.05; Supplementary Table 2). No significant between-profile differences were observed in time after stroke, place of residence, family history of stroke, regular physical exercise, smoking status, or drinking status. Although time after stroke was not statistically significant in the univariate analysis, it was included in the subsequent multivariable analysis as a clinically relevant covariate.

### 5.4 Multinomial logistic regression analysis of factors associated with latent profile membership

Multinomial logistic regression was performed with latent profile membership as the dependent variable. No substantial multicollinearity was detected among the variables included in the model, with all adjusted GVIF values below 2. The regression results are presented in Supplementary Table 4. Using the low supportive care needs group (Class 1) as the reference category, older age, anxiety score ≥5, and depression score ≥8 were associated with higher odds of membership in the moderate supportive care needs group (Class 2). Specifically, each additional year of age was associated with a 7.8% increase in the odds of being classified into Class 2 rather than Class 1 (OR = 1.078, 95% CI: 1.019–1.141, *p* = 0.009). Compared with participants with anxiety scores <5, those with anxiety scores ≥5 had higher odds of belonging to Class 2 (OR = 2.644, 95% CI: 1.429–4.892, *p* = 0.002). Similarly, compared with participants with depression scores <8, those with depression scores ≥8 were more likely to belong to Class 2 (OR = 2.569, 95% CI: 1.190–5.543, *p* = 0.016). In contrast, compared with participants with BI scores ≤40, those with BI scores of 41–60 (OR = 0.204, 95% CI: 0.072–0.582, *p* = 0.003) and 61–99 (OR = 0.126, 95% CI: 0.036–0.441, *p* = 0.001) had lower odds of being classified into Class 2. Participants with an above-high-school educational level were also less likely to belong to Class 2 than those with primary school education or below (OR = 0.299, 95% CI: 0.112–0.796, *p* = 0.016).

When comparing the high supportive care needs group (Class 3) with the low supportive care needs group (Class 1), older age, anxiety score ≥5, and depression score ≥8 were associated with higher odds of Class 3 membership. Each additional year of age was associated with an 11.7% increase in the odds of belonging to Class 3 rather than Class 1 (OR = 1.117, 95% CI: 1.052–1.186, *p* < 0.001). Compared with participants with anxiety scores <5, those with anxiety scores ≥5 had higher odds of being classified into Class 3 (OR = 2.481, 95% CI: 1.246–4.937, *p* = 0.010). Similarly, participants with depression scores ≥8 were more likely to belong to Class 3 than those with depression scores <8 (OR = 3.332, 95% CI: 1.472–7.546, *p* = 0.004). Compared with participants with BI scores ≤40, those with BI scores of 41–60 (OR = 0.179, 95% CI: 0.061–0.526, *p* = 0.002) and 61–99 (OR = 0.034, 95% CI: 0.007–0.173, *p* < 0.001) had lower odds of being classified into Class 3. Compared with participants with primary school education or below, those with junior/high school education (OR = 0.321, 95% CI: 0.154–0.668, *p* = 0.002) and above-high-school education (OR = 0.213, 95% CI: 0.072–0.627, *p* = 0.005) were less likely to belong to Class 3. In addition, participants with a monthly income ≥6,000 CNY had lower odds of Class 3 membership than those with a monthly income <4,000 CNY (OR = 0.253, 95% CI: 0.087–0.734, *p* = 0.011). No other variables remained statistically significant after adjustment.

## 6 Discussion

In this hospital-based cross-sectional study of older adults during early recovery after first-ever ischemic stroke, we identified three severity-graded profiles of supportive care needs: low, moderate, and high. The profiles primarily differed in overall need intensity rather than in qualitatively distinct configurations. Disease-related information needs were relatively prominent across all profiles, whereas life care, symptom management, and rehabilitation care needs were particularly elevated in the high-need profile. Older age, lower functional independence, anxiety symptoms, and depressive symptoms were cross-sectionally associated with membership in both the moderate- and high-need profiles. Educational attainment was also associated with profile membership, while the highest income category was associated with lower odds of membership in the high-need profile. The co-occurrence of greater need intensity with functional limitations, psychological symptoms, and fewer socioeconomic resources may be relevant to the planning of transitional care and follow-up services for older adults recovering from stroke.

The identification of three severity-graded profiles is broadly consistent with previous evidence indicating that the intensity of post-stroke care needs is not uniformly distributed across patients (13, 26). In the present study, however, the profiles primarily reflected graded differences in overall supportive care need intensity rather than clearly distinct need structures. Older adults across the three profiles therefore appeared to share a broadly similar configuration of supportive care needs while differing in the extent to which these needs were experienced. Accordingly, the profiles should not be interpreted as qualitatively distinct clinical phenotypes. Nevertheless, the moderate- and high-need profiles descriptively represent groups with greater need intensity and may provide a basis for further evaluation of tiered needs assessment strategies (27).

A notable finding was that disease-related information needs were among the most prominent domains across all three profiles. Although the present study assessed the intensity of disease-related information needs rather than whether those needs had been met, this finding is consistent with previous evidence that unmet informational needs are common after stroke and may persist beyond the acute phase (28). Stroke survivors may require continuing guidance regarding the nature of stroke, treatment plans, medication use, recurrence prevention, rehabilitation expectations, and home management (29). One possible explanation for the prominence of this domain is that some older adults may experience difficulties in health literacy, memory, or information processing, which could affect their ability to understand and apply complex medical and rehabilitation information (30). These mechanisms were not directly assessed in the present study. Nevertheless, the findings support consideration of disease-related information as a core component of supportive care assessment (27). Repeated, tailored, and comprehensible information provision during hospitalization and after discharge may therefore warrant further development and evaluation (31).

The high-need profile was characterized not only by elevated disease-related information needs but also by higher life care, symptom management, and rehabilitation care needs. This pattern is consistent with previous stroke research showing that poorer functional status and greater symptom burden tend to coexist with broader care needs (32). Older adults with greater need intensity may experience concurrent difficulties in daily living, symptom management, and rehabilitation participation. These difficulties could also increase the relevance of caregiver and professional support, although actual support use and caregiver involvement were not assessed in the present study (33). Patients assigned to the high-need profile may therefore warrant more comprehensive assessment of daily living, symptom management, rehabilitation, and post-discharge support needs (27). At the same time, the relative prominence of disease-related information needs in the low-need profile indicates that lower overall need intensity should not be interpreted as the absence of domain-specific supportive care needs.

Age and functional independence in activities of daily living were independently associated with profile membership. Older participants had higher odds of membership in the moderate- and high-need profiles, consistent with previous evidence that advancing age may be accompanied by more complex recovery and care needs after stroke (34). This association may reflect age-related recovery complexity that was not fully captured by the functional and clinical variables available in the present study (35). Higher Barthel Index categories were associated with lower odds of membership in both higher-need profiles, which is consistent with evidence linking functional dependence to greater care needs after stroke (36). Patients with lower functional independence may experience greater difficulty in daily activities and participation and may require more practical and rehabilitation-related support (37). These findings support the inclusion of functional status when conducting supportive care needs assessments and suggest that functional limitations should be considered when planning transitional and rehabilitation support (38). However, the magnitude of these associations should be interpreted cautiously because some combinations of functional category and profile membership contained relatively few participants, which may have reduced the precision and stability of the estimates.

Educational attainment was associated with both moderate- and high-need profile membership, although the pattern differed across educational categories. In contrast, the income association was limited to the comparison between the highest and lowest income categories for the high-need profile. One possible explanation is that educational attainment may be related to health literacy and the ability to understand and use health information (39), although health literacy was not directly measured in this study. Higher income may also facilitate access to rehabilitation, transportation, caregiving, or community resources (40), but these potential pathways were not assessed, and the present study measured need intensity rather than unmet need or service access. The findings are compatible with the possibility that fewer socioeconomic resources may coexist with greater supportive care need intensity (41, 42), but the underlying mechanisms cannot be determined from the present data. The associations were more consistently observed in comparisons involving the high-need profile, although the study did not formally test whether socioeconomic associations differed in magnitude between profiles. From an aging and public health perspective, these results support further investigation of how health literacy, service accessibility, and economic resources shape supportive care needs during stroke recovery.

Anxiety and depressive symptoms were cross-sectionally associated with membership in both the moderate- and high-need profiles. This finding is consistent with previous stroke research showing that emotional distress commonly coexists with greater care needs (43) and adverse recovery experiences (44). Patients experiencing psychological symptoms may report greater needs for reassurance, information, emotional support, and continuing professional care (45). Psychological symptoms may also coexist with lower confidence or reduced rehabilitation engagement (11), although these pathways were not assessed in the present study. Importantly, the observed associations may partly reflect the conceptual proximity between anxiety and depressive symptoms and the psychological care domain included in the supportive care needs measure, as well as their concurrent assessment using self-report instruments. The direction of association cannot be determined from the cross-sectional data: psychological symptoms may accompany greater perceived need intensity, while extensive care needs may also contribute to emotional distress. The findings should therefore be interpreted as evidence of concurrent association rather than causation. They support consideration of psychological symptom assessment alongside supportive care needs assessment in older adults recovering from stroke (46), while the effects of integrated psychological support on subsequent care needs require prospective evaluation.

Time after stroke was not independently associated with profile membership. However, participants were concentrated within a relatively narrow early-recovery period, despite the broader eligibility window of 15 days to 6 months. The absence of a statistically significant association should therefore not be interpreted as evidence that supportive care needs remain stable throughout recovery. Longitudinal studies with broader representation across recovery stages are needed to examine profile stability and transitions over time. Several other characteristics that differed across profiles in univariate analyses were not independently associated with profile membership after multivariable adjustment. These non-significant findings should not be interpreted as evidence of no association, particularly because some categories contained relatively few participants and several confidence intervals were wide. Accordingly, the absence of independent associations for body mass index, living arrangement, and number of chronic diseases should be interpreted cautiously rather than as evidence that these characteristics are unrelated to supportive care needs.

From an aging and public health perspective, the findings may have implications for the organization of supportive care during the transition from hospital treatment to rehabilitation, family care, and community follow-up. Within the local post-stroke care context of the study setting, supportive care extends beyond hospital-based treatment to rehabilitation and follow-up in family and community settings. Differences in the availability and accessibility of post-discharge rehabilitation and supportive services may therefore be relevant when interpreting patients’ continuing needs during recovery. The three profiles should be interpreted as descriptive patterns rather than as a formal classification tool and should not currently be used as fixed thresholds for individual triage or resource allocation. Disease-related information needs may warrant assessment across all patients, whereas those reporting greater overall need intensity may require more detailed evaluation of daily living, symptom management, rehabilitation, psychological, and post-discharge support needs. Age, functional status, psychological symptoms, educational attainment, and income may provide contextual information during assessment, but these characteristics should not substitute for directly assessing patients’ supportive care needs. As service availability, accessibility, and utilization were not directly assessed, these contextual considerations should not be interpreted as causal explanations for profile membership. Future studies should determine whether tiered assessment and follow-up strategies based on need intensity are feasible, acceptable, and effective within local hospital-to-community care pathways.

Several limitations should be acknowledged. First, the cross-sectional design precludes causal and temporal inference and does not allow assessment of profile stability or transitions over time. Second, participants were recruited by convenience sampling from three tertiary hospitals in one region of China and were concentrated in the early recovery period; therefore, the findings may not generalize to community-dwelling or long-term stroke survivors, patients with recurrent or hemorrhagic stroke, or those with severe cognitive, communication, psychiatric, or medical conditions. Third, supportive care needs, anxiety, and depressive symptoms were assessed concurrently using self-report measures, which may have introduced response and common method bias. Conceptual overlap between psychological symptoms and the psychological care domain may also have contributed to the observed associations. In addition, the scale measured need intensity rather than whether needs were unmet or whether services were accessible. Fourth, several potentially relevant factors, including stroke severity, lesion characteristics, cognitive function, frailty, rehabilitation exposure, health literacy, caregiver support, and service access, were not assessed, and residual confounding is therefore possible. Finally, profile membership was analyzed using most-likely class assignment rather than a three-step approach, the three-profile solution has not been externally replicated, and the profiles mainly reflected differences in overall need intensity.

## 7 Conclusion

Older ischemic stroke survivors during recovery exhibited severity-graded heterogeneity in supportive care needs, with three latent profiles characterized by low, moderate, and high overall levels of need. Disease-related information needs were prominent across all profiles, whereas life care, symptom management, and rehabilitation care needs were particularly elevated in the high-need profile. Membership in the higher-need profiles was cross-sectionally associated with older age, poorer self-care ability, anxiety symptoms, depressive symptoms, and lower educational attainment, while higher income was associated with lower odds of membership in the high-need profile. These findings provide descriptive evidence for needs-based assessment and the future development of tiered rehabilitation and follow-up support during stroke recovery. Further longitudinal and external validation is needed to examine the stability and potential application of these profiles in post-stroke care.

## Data Availability

The raw data supporting the conclusions of this article will be made available by the authors, without undue reservation.
