# Supplementary material for: Severity-graded supportive care need profiles in older adults during recovery after ischemic stroke: a latent profile analysis
Source: Front Public Health. 2026 Sep 14;14:1940952. doi: 10.3389/fpubh.2026.1940952 (PMC13618314; doi:10.3389/fpubh.2026.1940952)
Supplement: Supplementary file 1 [file Table_1.docx]

| **Section** | **Item No.** | **Recommendation** | **Location where item is reported** | **Relevant text from manuscript** |
| --- | --- | --- | --- | --- |
| Title and abstract | 1(a) | Indicate the study’s design with a commonly used term in the title or the abstract | Title and abstract | The title identifies the latent profile analysis. The abstract Methods explicitly states: “A cross-sectional study was conducted among 388 older adults with ischemic stroke recruited from three tertiary hospitals in China.” |
| Title and abstract | 1(b) | Provide in the abstract an informative and balanced summary of what was done and what was found | Title and abstract | The structured abstract provides Background, Methods, Results, and Conclusions. It summarizes the setting and sample, measurement of supportive care needs, latent profile analysis, multinomial logistic regression, the three identified profiles, associated factors, and the principal conclusion, together with the need for longitudinal and external validation. |
| Introduction | | | | |
| Background/rationale | 2 | Explain the scientific background and rationale for the investigation being reported | Introduction | The Introduction describes the public health burden of ischemic stroke in older adults, the multidimensional nature and heterogeneity of supportive care needs during recovery, limitations of variable-centered research, and the rationale for using a person-centered latent profile approach to inform needs-based assessment and follow-up care. |
| Objectives | 3 | State specific objectives, including any prespecified hypotheses | Introduction | The study aimed to identify latent profiles of supportive care needs among older adults during early recovery after first-ever ischemic stroke and to examine cross-sectional associations between participant characteristics and profile membership. No prespecified hypothesis was stated. |
| Methods | | | | |
| Study design | 4 | Present key elements of study design early in the paper | Methods: Study design and participants | The Methods section begins by identifying the investigation as a cross-sectional study using convenience sampling from three tertiary Grade A hospitals and states that reporting followed the STROBE statement for cross-sectional studies. |
| Setting | 5 | Describe the setting, locations, and relevant dates, including periods of recruitment, exposure, follow-up, and data collection | Methods: Study design and participants; Pilot study and data collection | Participants were recruited from neurology departments of three tertiary Grade A hospitals affiliated with the University of South China in Hengyang, Hunan Province, China, between November 2025 and March 2026. A pilot survey was conducted in November 2025, followed by formal data collection in the same hospital setting. |
| Participants | 6(a) | Cross-sectional study—Give the eligibility criteria, and the sources and methods of selection of participants | Methods: Study design and participants | Participants were selected by convenience sampling. Eligibility required age ≥60 years, first-ever ischemic stroke confirmed by CT or MRI according to Chinese guidelines, recovery phase of 15 days to 6 months with stable vital signs, ability to understand and respond to the questionnaire, and written informed consent. Exclusion criteria and the standardized recruitment and questionnaire procedures are also described. |
| Participants | 6(b) | Cohort study—For matched studies, give matching criteria and number of exposed and unexposed; Case-control study—For matched studies, give matching criteria and the number of controls per case | Not applicable | Not applicable. This was a cross-sectional study and did not use matched cohort or case-control sampling. |
| Variables | 7 | Clearly define all outcomes, exposures, predictors, potential confounders, and effect modifiers. Give diagnostic criteria, if applicable | Methods: Study design and participants; Measures | The manuscript defines the study population and ischemic stroke criteria; demographic, socioeconomic, clinical, functional, and psychological variables; supportive care need domain scores; latent profile membership; and the candidate predictors entered into multinomial logistic regression. No effect modifiers were prespecified. |
| Data sources/measurement | 8* | For each variable of interest, give sources of data and details of methods of assessment (measurement). Describe comparability of assessment methods if there is more than one group | Methods: Measures; Pilot study and data collection | Data were obtained using a self-designed demographic and clinical questionnaire, measured height and weight, self-reported or physician-diagnosed health characteristics, the Stroke Supportive Care Needs Scale, GDS-15, GAD-7, and Barthel Index. Scoring, cut-offs, reliability estimates, investigator assistance, and data checking procedures are reported. The same assessment procedures were used for all participants. |
| Bias | 9 | Describe any efforts to address potential sources of bias | Methods: Pilot study and data collection; Discussion: Limitations | Potential bias was addressed through a pilot study, trained investigators, standardized and neutral explanation of questionnaire items, recording participants’ own responses without prompting, on-site completeness checks, and independent verification of data entry. Harman’s single-factor test was used to assess common method bias. The Discussion also acknowledges selection, self-report, common method, and residual confounding concerns. |
| Study size | 10 | Explain how the study size was arrived at | Methods: Study design and participants | The sample size was based primarily on methodological recommendations for latent profile analysis. At least 300 participants were considered necessary for stable classification; allowing for 10%-20% incomplete or invalid questionnaires yielded a target of 330-375 participants. The final analytic sample was 388. |
| Quantitative variables | 11 | Explain how quantitative variables were handled in the analyses. If applicable, describe which groupings were chosen and why | Methods: Measures; Statistical analysis | Age, BMI, and time after stroke were analyzed as continuous variables. Categorical boundaries were specified for education, marital status, income, residence, living arrangement, family history, exercise, smoking, drinking, chronic disease count, anxiety, depression, and Barthel Index. The Barthel Index categories were based on previous stroke supportive care needs research. Living arrangement was dichotomized for regression because the nursing-home and living-alone groups were small. |
| Statistical methods | 12(a) | Describe all statistical methods, including those used to control for confounding | Methods: Statistical analysis | Analyses included descriptive statistics, Harman’s single-factor test, one- to five-profile latent profile models, model selection using AIC, BIC, aBIC, LMR-LRT, BLRT, entropy, posterior probabilities, class size and interpretability, between-profile comparisons, multinomial logistic regression, and GVIF assessment. Covariates were selected using univariate significance, clinical relevance, prior evidence, and avoidance of redundant or outcome-proximal variables. ORs and 95% CIs were reported. |
| Statistical methods | 12(b) | Describe any methods used to examine subgroups and interactions | Methods: Statistical analysis | No prespecified interaction analyses were performed. Analytic groups were the latent profiles identified by LPA; demographic, clinical, functional, and psychological characteristics were compared across these profiles using ANOVA, Kruskal-Wallis, chi-square, or Fisher’s exact tests, as appropriate. |
| Statistical methods | 12(c) | Explain how missing data were addressed | Methods: Pilot study and data collection | After on-site checks and clarification with participants, no item-level missing data remained. All 388 participants had complete data for variables in the main analyses; no imputation was conducted and no participant was excluded because of missing data. |
| Statistical methods | 12(d) | Cohort study—If applicable, explain how loss to follow-up was addressed; Case-control study—If applicable, explain how matching of cases and controls was addressed; Cross-sectional study—If applicable, describe analytical methods taking account of sampling strategy | Methods: Study design and participants; Statistical analysis | Not applicable to cohort or case-control designs. The cross-sectional study used convenience sampling from three hospitals. No complex sampling design, sampling weights, matching, or cluster-adjusted analysis was used. |
| Statistical methods | 12(e) | Describe any sensitivity analyses | Not applicable | No formal sensitivity analysis was reported. |
| Results | | | | |
| Participants | 13(a)* | Report numbers of individuals at each stage of study—e.g., numbers potentially eligible, examined for eligibility, confirmed eligible, included in the study, completing follow-up, and analyzed | Results: Participants | A total of 388 participants were included and analyzed. The manuscript states that the participant selection process and numbers at each stage are presented in Supplementary Figure 2. |
| Participants | 13(b)* | Give reasons for non-participation at each stage | Results: Participants | The main manuscript refers readers to Supplementary Figure 2 for the participant selection process; reasons for non-participation or exclusion are not listed in the main text. |
| Participants | 13(c)* | Consider use of a flow diagram | Results: Participants | A participant flow diagram is provided as Supplementary Figure 2. |
| Descriptive data | 14(a)* | Give characteristics of study participants (e.g., demographic, clinical, social) and information on exposures and potential confounders | Results: Participants; Supplementary Table 2 | The Results report a median age of 70.00 years (IQR 65.00-75.00), 54.4% male participants, and a median time after stroke of 18.00 days (IQR 17.00-24.00). Detailed demographic, socioeconomic, clinical, functional, and psychological characteristics overall and by profile are provided in Supplementary Table 2. |
| Descriptive data | 14(b)* | Indicate number of participants with missing data for each variable of interest | Methods: Pilot study and data collection; Results: Participants | No item-level missing data remained in the final dataset, and all 388 participants had complete data for the variables included in the main analyses. |
| Descriptive data | 14(c)* | Cohort study—Summarize follow-up time (e.g., average and total amount) | Not applicable | Not applicable. This was a cross-sectional study and did not include follow-up. |
| Outcome data | 15* (cohort) | Cohort study—Report numbers of outcome events or summary measures over time | Not applicable | Not applicable. This was not a cohort study. |
| Outcome data | 15* (case-control) | Case-control study—Report numbers in each exposure category, or summary measures of exposure | Not applicable | Not applicable. This was not a case-control study. |
| Outcome data | 15* (cross-sectional) | Cross-sectional study—Report numbers of outcome events or summary measures | Results: Participants; Latent Profile Analysis; Supplementary Tables 2-4 | Cross-sectional summary measures are reported for participant characteristics, supportive care need domain scores, profile frequencies, and associated factors. Three profiles were identified: low supportive care needs (n = 120, 30.9%), moderate supportive care needs (n = 142, 36.6%), and high supportive care needs (n = 126, 32.5%). |
| Main results | 16(a) | Give unadjusted estimates and, if applicable, confounder-adjusted estimates and their precision (e.g., 95% confidence interval). Make clear which confounders were adjusted for and why they were included | Results: Univariate analysis and multinomial logistic regression analysis; Supplementary Tables 2 and 4 | Unadjusted between-profile comparisons are presented in Supplementary Table 2. Adjusted multinomial logistic regression estimates are presented in Supplementary Table 4 as ORs with 95% CIs, using the low-need profile as the reference. The manuscript explains that candidate covariates were selected using univariate results, clinical relevance, prior evidence, and avoidance of redundant or outcome-proximal variables. |
| Main results | 16(b) | Report category boundaries when continuous variables were categorized | Methods: Measures; Results: Multinomial logistic regression analysis | The manuscript reports all category boundaries, including income (<4000, 4000-5999, and ≥6000 CNY), chronic disease count (0, 1-2, and ≥3), anxiety (GAD-7 <5 vs ≥5), depression (GDS-15 <8 vs ≥8), and Barthel Index (≤40, 41-60, and 61-99). Education and other categorical variables are also explicitly defined; age, BMI, and time after stroke were continuous. |
| Main results | 16(c) | If relevant, consider translating estimates of relative risk into absolute risk for a meaningful time period | Not applicable | Not applicable. The study did not estimate relative risks over time; multinomial logistic regression results are reported as odds ratios with 95% confidence intervals. |
| Other analyses | 17 | Report other analyses done—e.g., analyses of subgroups and interactions, and sensitivity analyses | Results: Assessment of Common Method Bias; Latent Profile Analysis; Univariate analysis; Multinomial logistic regression analysis | Other analyses included Harman’s single-factor test, comparison of one- to five-profile solutions using multiple fit and classification indices, assessment of average posterior probabilities, univariate comparisons across profiles, and GVIF assessment before multinomial regression. No subgroup, interaction, or formal sensitivity analyses were reported. |
| Discussion | | | | |
| Key results | 18 | Summarize key results with reference to study objectives | Discussion: Key results | The opening Discussion paragraph summarizes the three severity-graded supportive care need profiles, the prominence of disease-related information needs, the elevated daily living, symptom management, and rehabilitation needs in the high-need profile, and the demographic, functional, psychological, educational, and income factors associated with profile membership. |
| Limitations | 19 | Discuss limitations of the study, taking into account sources of potential bias or imprecision. Discuss both direction and magnitude of any potential bias | Discussion: Limitations | Limitations include the cross-sectional design; convenience sampling from three tertiary hospitals in one region; concentration in early recovery; restricted generalizability to excluded clinical groups; concurrent self-report measurement and possible common method or response bias; conceptual overlap between psychological symptoms and psychological care needs; measurement of need intensity rather than unmet need; unmeasured confounding; use of most-likely class assignment; and lack of external replication. The manuscript also notes small cells and wide confidence intervals that may reduce estimate precision and stability. |
| Interpretation | 20 | Give a cautious overall interpretation of results considering objectives, limitations, multiplicity of analyses, results from similar studies, and other relevant evidence | Discussion: Interpretation | The Discussion interprets the profiles as descriptive severity patterns rather than fixed clinical phenotypes or a validated triage tool. Associations are described as cross-sectional and non-causal, alternative explanations and measurement overlap are considered, non-significant findings are interpreted cautiously, and longitudinal and external validation are recommended. |
| Generalisability | 21 | Discuss the generalisability (external validity) of the study results | Discussion: Limitations | External validity is addressed in the limitations: recruitment from three tertiary hospitals in one region, convenience sampling, concentration in early recovery, and exclusion of recurrent or hemorrhagic stroke and participants with severe cognitive, communication, psychiatric, or medical conditions may limit generalizability to community-dwelling, long-term, or clinically more complex stroke populations. |
| Other information | | | | |
| Funding | 22 | Give the source of funding and the role of the funders for the present study and, if applicable, for the original study on which the present article is based | Declarations: Funding | The Funding section lists the funding programs and grant numbers supporting the study. A separate statement describing the role of the funders in study design, data collection, analysis, interpretation, or manuscript preparation is not included. |

**Give information separately for cases and controls in case-control studies and, if applicable, for exposed and unexposed groups in cohort and cross-sectional studies.*

Note: An Explanation and Elaboration article discusses each checklist item and gives methodological background and published examples of transparent reporting. Information on the STROBE Initiative is available at www.strobe-statement.org.

**Supplementary Table 2. Demographic and clinical characteristics of participants across latent profiles (N = 388)**

| Variables | Overall | Class 1 | Class 2 | Class 3 | Statistic | P value |
| --- | --- | --- | --- | --- | --- | --- |
| N | 388 | 120 | 142 | 126 |  |  |
| Age, years^†^ | 70.00 (65.00, 75.00) | 68.00 (63.00, 71.00) | 71.00 (66.00, 74.75) | 73.50 (67.00, 79.00) | 47.928 | <0.001 |
| BMI, kg/m²^†^ | 23.45 (22.23, 24.56) | 23.39 (22.82, 24.21) | 23.91 (22.61, 24.77) | 23.12 (21.50, 24.63) | 6.802 | 0.033 |
| Time after stroke, days^†^ | 18.00 (17.00, 24.00) | 19.00 (16.00, 23.25) | 18.00 (17.00, 23.00) | 18.00 (17.00, 25.00) | 0.228 | 0.892 |
| Sex^‡^ |  |  |  |  | 8.215 | 0.016 |
| Male | 211 (54.4%) | 77 (64.2%) | 76 (53.5%) | 58 (46.0%) |  |  |
| Female | 177 (45.6%) | 43 (35.8%) | 66 (46.5%) | 68 (54.0%) |  |  |
| Educational level^‡^ |  |  |  |  | 17.622 | 0.001 |
| Primary school and below | 164 (42.3%) | 36 (30.0%) | 58 (40.8%) | 70 (55.6%) |  |  |
| Junior/high school | 165 (42.5%) | 60 (50.0%) | 65 (45.8%) | 40 (31.7%) |  |  |
| Above high school | 59 (15.2%) | 24 (20.0%) | 19 (13.4%) | 16 (12.7%) |  |  |
| Marital status^‡^ |  |  |  |  | 11.442 | 0.003 |
| Unmarried/divorced/widowed | 71 (18.3%) | 15 (12.5%) | 21 (14.8%) | 35 (27.8%) |  |  |
| Married | 317 (81.7%) | 105 (87.5%) | 121 (85.2%) | 91 (72.2%) |  |  |
| Monthly income^‡^ |  |  |  |  | 23.973 | <0.001 |
| <4000 CNY | 68 (17.5%) | 10 (8.3%) | 26 (18.3%) | 32 (25.4%) |  |  |
| 4000–5999 CNY | 177 (45.6%) | 56 (46.7%) | 55 (38.7%) | 66 (52.4%) |  |  |
| ≥6000 CNY | 143 (36.9%) | 54 (45.0%) | 61 (43.0%) | 28 (22.2%) |  |  |
| Place of residence^‡^ |  |  |  |  | 1.207 | 0.547 |
| Rural | 76 (19.6%) | 20 (16.7%) | 28 (19.7%) | 28 (22.2%) |  |  |
| Urban | 312 (80.4%) | 100 (83.3%) | 114 (80.3%) | 98 (77.8%) |  |  |
| Living arrangement^§^ |  |  |  |  |  | 0.004 |
| Living with family/others | 330 (85.1%) | 111 (92.5%) | 120 (84.5%) | 99 (78.6%) |  |  |
| Nursing home | 27 (7.0%) | 2 (1.7%) | 8 (5.6%) | 17 (13.5%) |  |  |
| Living alone | 31 (8.0%) | 7 (5.8%) | 14 (9.9%) | 10 (7.9%) |  |  |
| Family history of stroke^‡^ |  |  |  |  | 0.105 | 0.949 |
| No | 255 (65.7%) | 79 (65.8%) | 92 (64.8%) | 84 (66.7%) |  |  |
| Yes | 133 (34.3%) | 41 (34.2%) | 50 (35.2%) | 42 (33.3%) |  |  |
|  |  |  |  |  |  |  |

**Supplementary Table 2. Continued**

| Variables | Overall | Class 1 | Class 2 | Class 3 | Statistic | P value |
| --- | --- | --- | --- | --- | --- | --- |
| Drinking status^‡^ |  |  |  |  | 1.320 | 0.517 |
| No/former | 317 (81.7%) | 94 (78.3%) | 118 (83.1%) | 105 (83.3%) |  |  |
| Current | 71 (18.3%) | 26 (21.7%) | 24 (16.9%) | 21 (16.7%) |  |  |
| Self-care ability, BI score^§^ |  |  |  |  |  | <0.001 |
| ≤40 | 112 (28.9%) | 5 (4.2%) | 47 (33.1%) | 60 (47.6%) |  |  |
| 41–60 | 229 (59.0%) | 86 (71.7%) | 80 (56.3%) | 63 (50.0%) |  |  |
| 61-99 | 47 (12.1%) | 29 (24.2%) | 15 (10.6%) | 3 (2.4%) |  |  |
| Number of chronic diseases^§^ |  |  |  |  |  | <0.001 |
| 0 | 34 (8.8%) | 21 (17.5%) | 9 (6.3%) | 4 (3.2%) |  |  |
| 1–2 | 238 (61.3%) | 75 (62.5%) | 89 (62.7%) | 74 (58.7%) |  |  |
| ≥3 | 116 (29.9%) | 24 (20.0%) | 44 (31.0%) | 48 (38.1%) |  |  |
| Anxiety symptoms^‡^ |  |  |  |  | 30.262 | <0.001 |
| <5 | 207 (53.4%) | 89 (74.2%) | 63 (44.4%) | 55 (43.7%) |  |  |
| ≥5 | 181 (46.6%) | 31 (25.8%) | 79 (55.6%) | 71 (56.3%) |  |  |
| Depressive symptoms^‡^ |  |  |  |  | 33.969 | <0.001 |
| <8 | 255 (65.7%) | 103 (85.8%) | 87 (61.3%) | 65 (51.6%) |  |  |
| ≥8 | 133 (34.3%) | 17 (14.2%) | 55 (38.7%) | 61 (48.4%) |  |  |
| Regular physical exercise^‡^ |  |  |  |  | 3.642 | 0.162 |
| Yes | 179 (46.1%) | 62 (51.7%) | 57 (40.1%) | 60 (47.6%) |  |  |
| No | 209 (53.9%) | 58 (48.3%) | 85 (59.9%) | 66 (52.4%) |  |  |
| Smoking status^‡^ |  |  |  |  | 2.748 | 0.253 |
| No/former | 299 (77.1%) | 92 (76.7%) | 104 (73.2%) | 103 (81.7%) |  |  |
| Current | 89 (22.9%) | 28 (23.3%) | 38 (26.8%) | 23 (18.3%) |  |  |

**Note.** Data are presented as median (interquartile range) for non-normally distributed continuous variables and n (%) for categorical variables. Variables marked with †, ‡, and § were compared using the Kruskal-Wallis test, chi-square test, and Fisher's exact test, respectively. The Statistic column reports H values for Kruskal-Wallis tests and chi-square statistics for chi-square tests; for Fisher's exact test, only P values are reported. BMI, body mass index; BI, Barthel Index.

**Supplementary Table 3. Domain scores of supportive care needs across the three latent profiles**

| Dimension | Overall | Class 1 | Class 2 | Class 3 |
| --- | --- | --- | --- | --- |
| Life care needs | 1.66 ± 0.85 | 0.77 ± 0.43 | 1.58 ± 0.48 | 2.60 ± 0.37 |
| Symptom management needs | 1.94 ± 0.91 | 0.81 ± 0.42 | 2.18 ± 0.52 | 2.75 ± 0.40 |
| Rehabilitation care needs | 1.62 ± 0.77 | 0.82 ± 0.37 | 1.59 ± 0.45 | 2.43 ± 0.43 |
| Disease-related information needs | 2.35 ± 0.76 | 1.52 ± 0.60 | 2.60 ± 0.50 | 2.86 ± 0.41 |
| Psychological care needs | 1.07 ± 0.62 | 0.59 ± 0.34 | 0.96 ± 0.46 | 1.64 ± 0.52 |
| Social support needs | 1.16 ± 0.63 | 0.64 ± 0.44 | 1.13 ± 0.46 | 1.68 ± 0.53 |

**Supplementary Table 4. Results of multinomial logistic regression (N=388)**

| Variables | Class 2 | | | Class 3 | | |
| --- | --- | --- | --- | --- | --- | --- |
|  | β | P | OR (95% CI) | β | P | OR (95% CI) |
| Age, years | 0.075 | 0.009 | 1.078 (1.019, 1.141) | 0.111 | <0.001 | 1.117 (1.052, 1.186) |
| BMI, kg/m² | 0.053 | 0.446 | 1.055 (0.920, 1.209) | -0.008 | 0.916 | 0.992 (0.858, 1.147) |
| Time after stroke, days | -0.012 | 0.695 | 0.989 (0.933, 1.047) | -0.000 | 0.997 | 1.000 (0.939, 1.064) |
| Sex |  |  |  |  |  |  |
| Female | 0.269 | 0.376 | 1.309 (0.721, 2.378) | 0.487 | 0.143 | 1.628 (0.848, 3.124) |
| Educational level |  |  |  |  |  |  |
| Junior/high school | -0.624 | 0.065 | 0.536 (0.276, 1.039) | -1.137 | 0.002 | 0.321 (0.154, 0.668) |
| Above high school | -1.208 | 0.016 | 0.299 (0.112, 0.796) | -1.549 | 0.005 | 0.213 (0.072, 0.627) |
| Marital status |  |  |  |  |  |  |
| Married | 0.620 | 0.183 | 1.860 (0.746, 4.633) | 0.189 | 0.684 | 1.209 (0.485, 3.013) |
| Monthly income, CNY |  |  |  |  |  |  |
| 4000–5999 | -0.728 | 0.143 | 0.483 (0.182, 1.279) | -0.664 | 0.206 | 0.515 (0.184, 1.442) |
| ≥6000 | -0.655 | 0.185 | 0.519 (0.197, 1.368) | -1.373 | 0.011 | 0.253 (0.087, 0.734) |
| Living arrangement |  |  |  |  |  |  |
| Not living with family/others | 0.931 | 0.085 | 2.537 (0.879, 7.324) | 1.060 | 0.064 | 2.885 (0.939, 8.870) |
| Self-care ability, BI score |  |  |  |  |  |  |
| 41–60 | -1.589 | 0.003 | 0.204 (0.072, 0.582) | -1.721 | 0.002 | 0.179 (0.061, 0.526) |
| 61–99 | -2.068 | 0.001 | 0.126 (0.036, 0.441) | -3.395 | <0.001 | 0.034 (0.007, 0.173) |
| Number of chronic diseases |  |  |  |  |  |  |
| 1–2 | 0.498 | 0.317 | 1.646 (0.620, 4.368) | 0.937 | 0.163 | 2.552 (0.684, 9.526) |
| ≥3 | 0.353 | 0.532 | 1.423 (0.470, 4.310) | 0.822 | 0.256 | 2.276 (0.551, 9.399) |
| Anxiety symptoms |  |  |  |  |  |  |
| ≥5 | 0.972 | 0.002 | 2.644 (1.429, 4.892) | 0.909 | 0.010 | 2.481 (1.246, 4.937) |
| Depressive symptoms |  |  |  |  |  |  |
| ≥8 | 0.943 | 0.016 | 2.569 (1.190, 5.543) | 1.204 | 0.004 | 3.332 (1.472, 7.546) |

Note. Class 1 was used as the reference group. β, regression coefficient; OR, odds ratio; CI, confidence interval. Reference categories were male, primary school and below, unmarried/divorced/widowed, <4000 CNY, living with family/others, BI score ≤40, no chronic diseases, anxiety symptoms <5, and depressive symptoms <8. Living arrangement was dichotomized as living with family/others versus not living with family/others in the regression mod

**Supplementary Figure 1. Self-designed General Demographic and Clinical Characteristics Questionnaire**

**Supplementary Figure 2. Flow chart of participant selection**
